# Respiratory support in acute heart failure with preserved vs reduced ejection fraction

**DOI:** 10.1002/clc.23317

**Published:** 2019-12-11

**Authors:** Thomas S. Metkus, Robert Scott Stephens, Steven Schulman, Steven Hsu, David A. Morrow, Shaker M. Eid

**Affiliations:** ^1^ Division of Cardiology, Department of Medicine Johns Hopkins University School of Medicine Baltimore Maryland; ^2^ Division of Pulmonary and Critical Care Medicine, Department of Medicine Johns Hopkins University School of Medicine Baltimore Maryland; ^3^ Cardiovascular Division, Department of Medicine Brigham and Women's Hospital, Harvard Medical School Boston Massachusetts; ^4^ Department of Medicine Johns Hopkins University School of Medicine Baltimore Maryland

**Keywords:** acute coronary care, cardiac function, heart failure

## Abstract

**Background:**

There is little evidence addressing the use and differential impact of respiratory support in acute heart failure (AHF) patients with preserved (HFPEF) vs reduced (HFREF) ejection fraction. Therefore, our objective was to determine the usage and clinical outcomes of critical care respiratory support in AHF across the two populations.

**Hypothesis:**

Respiratory support would be associated with adverse outcome in both HFPEF and HFREF.

**Methods:**

We identified HFPEF, HFREF, invasive mechanical ventilation (IMV), and noninvasive ventilation (NIV) using International Classification of Disease‐Ninth Edition codes in the National Inpatient Sample between January 1, 2008 and December 31, 2014. We determined rates of IMV and NIV use. We identified predictors of need for IMV and NIV and the association between ventilation strategies and in‐hospital mortality in HFPEF vs HFREF.

**Results:**

1.3 million AHF‐HFPEF and 1.7 million AHF‐HFREF hospitalizations were included; 5.98% of AHF HFPEF hospitalizations included NIV and 0.57% included IMV. Among HFREF hospitalizations, fewer (4.1%) included NIV and more (0.93%) included IMV. In HFPEF hospitalization, NIV use was associated with 2.24‐fold increased risk for death compared to no respiratory support in an adjusted model (HR 2.24 95% CI 2.05‐2.44) and IMV use was associated with 2.85‐fold increased risk for death (HR 2.85 95% CI 2.30‐3.53). This increased risk of in‐hospital mortality was similar among HFREF patients.

**Conclusions:**

Use of respiratory support is increasing among patients with both HFPEF and HFREF and associated with substantially increased mortality in both heart failure subtypes.

## 1 INTRODUCTION

Acute heart failure (AHF) hospitalization is responsible for millions of hospital visits per year^1^ and substantial morbidity, mortality, and reduced quality of life.^2^ The heart failure epidemic involves both heart failure with preserved and reduced ejection fraction, and approximately half of patients with heart failure have preserved ejection fraction.^3^ Heart failure with preserved ejection fraction (HFPEF) and heart failure with reduced ejection fraction (HFREF) have important differences in pathophysiology,^4^ cause of death,^5^ and comorbidities.^6^ Specifically, patients with HFPEF frequently suffer comorbid pulmonary disease,^7^ skeletal muscle abnormalities,^8^ obstructive sleep apnea^9^ and obesity^10^, ^11^ and right ventricular involvement and pulmonary vascular disease^12^, ^13^, ^14^ which contribute to a syndrome distinct from that of HFREF.

AHF patients often require respiratory support secondary to pulmonary edema, reduced cardiac output, respiratory muscle fatigue, and pleural effusion and the burden of respiratory failure in AHF is increasing.^15^ Respiratory support can be provided with either noninvasive ventilation (NIV) with facemask or invasive mechanical ventilation (IMV) through an endotracheal tube.^16^, ^17^, ^18^ Need for respiratory support in AHF is increasing and associated with substantially worsened outcomes including mortality.^16^, ^19^, ^20^, ^21^ Yet, there is little evidence addressing the differential impact of respiratory failure or mode of respiratory support in AHF patients with HFPEF vs HFREF. Given the pathophysiologic differences between the two syndromes, the incidence, causes, and prognostic significance of respiratory failure in the two populations may differ. Moreover, there is little evidence addressing critical care needs in the HFEPF population, which is a requisite for planning randomized trials of support in this important population. Understanding differences in respiratory support in the two populations would inform intervention, risk stratification, and planning of future trials.

To address these gaps in knowledge, we conducted a nationwide retrospective cohort study to characterize the use of IMV and NIV in AHF subpopulations of HFPEF vs HFREF as well as to identify predictors of requirement for respiratory support and associated hazard for mortality in the two populations. We hypothesized that the epidemiology of usage of IMV and NIV would be different between the two diseases and the predictors of need for IMV and NIV would differ between HFPEF and HFREF patients. Finally, we hypothesized that need for respiratory support would be associated with adverse outcome in both HFPEF and HFREF.

## 2 METHODS

### 2.1 Study population

Our study population included AHF hospitalizations from the National Inpatient Sample (NIS) between January 1, 2008 and December 31, 2014. The NIS is a large all‐payer inpatient care database representative of US hospitals. The Institutional Review Board of Johns Hopkins University and the Healthcare Cost and Utilization Project approved the project.

All diagnoses and procedures in the NIS are identified by International Classification of Disease‐Ninth Edition‐ Clinical Modification (ICD‐9‐CM) codes. The first diagnosis listed is the primary reason for hospital admission. We included all hospitalizations with a principal diagnosis of AHF with HFPEF or HFREF based on ICD‐9 codes 428.31 or 428.33 for HFPEF and 428.21 or 428.23 for HFREF which have been validated for identification of heart failure admissions.^22^ ICD‐9 CM codes used for variable definition are shown in supplemental Table S1. We adhered to the NIS methods advocated by Khera et al.^23^

### 2.2 Exposures and outcomes

Our exposure of interest was receipt of IMV or NIV compared to no support in patients with HFPEF and HFREF. IMV use was identified using ICD‐9‐CM code 96.7x for mechanical ventilation or 96.0 for endotracheal intubation which are specific for mechanical ventilation.^21^, ^24^ NIV was identified with code 93.90.^25^ We considered only IMV and NIV within the first 24 hours because our clinical interest was respiratory failure related to initial AHF presentation rather than other factors such as surgery, pneumonia, or other hospital acquired condition. We used standard ICD‐9 codes and NIS‐provided Elixhauser comorbidities to define other clinical covariates of a priori clinical interest. A list of all ICD‐9 codes is displayed in Supplemental Table S1.

We first considered the outcome of use of IMV or NIV vs neither modality in both the HFPEF and HFREF populations. Thus, our primary outcome for an initial set of analyses was IMV or NIV use stratified by HFPEF vs HFREF. Then, to determine the association of respiratory support with outcome in each of HFPEF and HFREF, we divided each of the HFPEF and HFREF populations into three groups: AHF using IMV, AHF using NIV, and AHF using no ventilation. To assess the association of IMV and NIV use with outcome in the HFPEF and HFREF populations, we used a primary outcome of in‐hospital mortality within 30 days.

### 2.3 Statistical analysis

Clinical characteristics and outcomes were compared between groups with the Pearson χ^2^ test for categorical variables and one‐way analysis of variance for continuous variables. Annual rates of IMV and NIV use within HFPEF and HFREF were calculated per 1000 AHF patients during the study period. Multivariable regression models adjusting for age and sex were constructed according to mode of ventilation in the HFPEF and HFREF populations to identify changes in mortality over time, adjusting for clustering of patients within hospitals. To determine the predictors of need for IMV and NIV in each heart failure population, we performed survey‐weighted logistic regression models with IMV and NIV as dependent variables and adjusted for factors of a priori interest based on our conceptual model. To determine the association between ventilation strategies and in‐hospital mortality in HFPEF and HFREF, we performed univariable and adjusted Cox proportional hazards models censoring at hospital discharge or 30 days of hospital stay, whichever came first. Survival curves were generated with the Kaplan‐Meier method and compared using the log‐rank test. Analysis was performed using Stata/MP version 13.0 (StataCorp Inc., College Station, Texas). A two‐tailed *P* value less than .05 was considered statistically significant.

## 3 RESULTS

### 3.1 Use of respiratory support in HFPEF and HFREF

**Figure 1:**
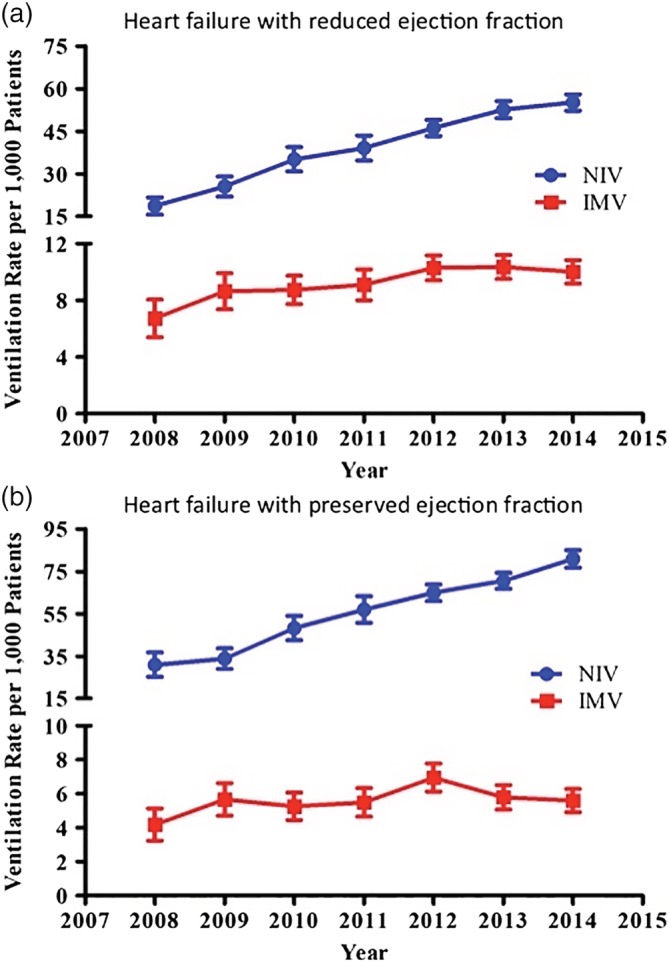
Usage of noninvasive and invasive ventilation in acute heart failure hospitalizations with reduced and preserved ejection fraction. *P* < .05 for all trends increasing over time

1 331 236 AHF‐HFPEF and 1 707 762 AHF‐HFREF hospitalizations were included. 5.98% of AHF‐HFPEF hospitalizations included NIV and 0.57% included IMV. Among HFREF hospitalizations, 4.1% included NIV and 0.93% included IMV. Rates of IMV and NIV use increased over the study period in both populations (Figure 1).

### 3.2 Clinical characteristics and outcomes of respiratory support in HFPEF and HFREF

Baseline characteristics of the study cohort are displayed in Table 1. HFPEF patients were older than HFREF patients, a higher percentage of HFPEF patients were female, and a higher percentage of HFPEF patients were of white race.

**Table 1:** Baseline characteristics of patients undergoing AHF hospitalization treated with respiratory support

(a) Baseline characteristics of patients with AHF due to HFPEF, NIS 2002‐2013						
					*P* value	
Characteristics	All (*n* = 1 331 236)	AHF without IMV or NIV (*n* = 1 244 078)	AHF with NIV (*n* = 79 571)	AHF with IMV (*n* = 7586)	NIV vs neither	IMV vs neither
Age, mean (95% CI)	75.9 (75.8‐76.1)	76.1 (76.0‐76.3)	73.7 (73.4‐74.0)	70.6 (69.9‐71.2)	<.001	<.001
Female sex, No. (%)	853 975 (64.1)	799 153 (64.2)	50 358 (63.3)	4464 (58.8)	.02	<.001
Race, No. (%)						
White	1 000 896 (75.1)	939 961 (75.6)	56 408 (70.9)	4527 (59.7)		
Black	216 556 (16.3)	199 588 (16.0)	14 949 (18.8)	2020 (26.6)	<.001	<.001
Hispanic	86 222 (6.5)	79 308 (6.4)	6136 (7.7)	778 (10.3)	<.001	<.001
Asian/Pacific Islander	21 664 (1.6)	19 691 (1.6)	1732 (2.2)	242 (3.2)	<.001	<.001
Native American	5898 (0.4)	5531 (0.4)	347 (0.4)	20.1 (0.3)	.8	.6
Comorbidities, No. (%)						
Chronic pulmonary disease	552 952 (41.5)	504 796 (40.6)	44 785 (56.3)	3371 (44.4)	<.001	.002
Chronic renal failure	578 850 (43.5)	538 419 (43.3)	36 927 (46.4)	3505 (46.2)	<.001	.02
Coronary artery disease	584 865 (43.9)	547 626 (44.0)	34 020 (42.8)	3219 (42.4)	.003	.2
Diabetes mellitus	619 221 (46.5)	572 669 (46.0)	42 575 (53.5)	3947 (52.0)	<.001	<.001
Dyslipidemia	601 653 (45.2)	562 381 (45.2)	36 304 (45.6)	2968 (39.1)	.4	<.001
Hypertension	1 054 714 (79.2)	986 558 (79.3)	62 613 (78.7)	5543 (73.0)	.1	<.001
Obesity	297 741 (22.4)	266 893 (21.5)	28 491 (35.8)	2358 (31)	<.001	<.001
Previous myocardial infarction	129 024 (9.7)	119 915 (9.6)	8384 (10.5)	725 (9.6)	<.001	.9
Previous CABG	169 429 (12.7)	161 148 (13.0)	7592 (9.5)	690 (9.1)	<.001	<.001
Smoking	310 247 (23.3)	285 442 (22.9)	23 168 (29.1)	1636 (21.6)	<.001	.2
Charlson comorbidity index, No. (%)					<.001	.5
1	200 267 (15.0)	191 484 (15.4)	7580 (9.5)	1203 (15.9)		
2	292 947 (22.0)	273 519 (22.0)	17 739 (22.3)	1688 (22.3)		
≥3	838 022 (63.0)	779 075 (62.6)	54 252 (68.2)	4695 (61.9)		

(b) Baseline characteristics of patients with AHF due to HFREF, NIS 2002‐2013						
					*P* value	
Characteristics	All (*n* = 1 707 762)	AHF without IMV or NIV (*n* = 1 621 523)	AHF with NIV (*n* = 70 287)	AHF with IMV (*n* = 15 952)	NIV vs neither	IMV vs neither
Age, mean (SD)	71.0 (70.8‐71.2)	71.0 (70.8‐71.2)	71.5 (71.3‐71.8)	67.1 (66.6‐67.6)	<.001	<.001
Female sex, No. (%)	677 557 (39.7)	640 827 (39.5)	30 041 (42.7)	6689 (41.9)	<.001	.006
Race, No. (%)						
White	1 159 065 (67.9)	1 101 913 (68.0)	47 111 (67.0)	10 041 (62.9)		
Black	387 837 (22.7)	368 561 (22.7)	15 399 (21.9)	3877 (24.3)	.5	.002
Hispanic	124 417 (7.3)	116 910 (7.2)	5956 (8.5)	1551 (9.7)	<.001	<.001
Asian/Pacific Islander	26 761 (1.6)	24 832 (1.5)	1537 (2.2)	392 (2.5)	<.001	<.001
Native American	9682 (0.6)	9307 (0.6)	284 (0.4)	91 (0.6)	.05	.8
Comorbidities, No. (%)						
Chronic pulmonary disease	592 495 (34.7)	551 959 (34.0)	34 861 (49.6)	5675 (35.6)	<.001	.07
Chronic renal failure	694 192 (40.6)	657 323 (40.5)	30 837 (43.9)	6032 (37.8)	<.001	.002
Coronary artery disease	1 024 960 (60.0)	972 493 (60.0)	43 321 (61.6)	9147 (57.3)	<.001	.003
Diabetes mellitus	747 512 (43.8)	706 355 (43.6)	34 504 (49.1)	6653 (41.7)	<.001	.04
Dyslipidemia	769 923 (45.1)	730 801 (45.1)	33 173 (47.2)	5949 (37.3)	<.001	<.001
Hypertension	1 248 282 (73.1)	1 184 318 (73.0)	53 505 (76.1)	10 459 (65.6)	<.001	<.001
Obesity	246 743 (14.4)	228 164 (14.1)	16 282 (23.2)	2298 (14.4)	<0.001	.6
Previous myocardial infarction	292 838 (17.1)	276 014 (17.0)	14 261 (20.3)	2563 (16.1)	<.001	.2
Previous CABG	326 866 (19.1)	313 491 (19.3)	11 325 (16.1)	2049 (12.8)	<.001	<.001
Smoking	482 745 (28.3)	454 608 (28.0)	23 497 (33.4)	4640 (29.1)	<.001	.2
Charlson comorbidity index, No. (%)					<.001	<.001
1	289 801 (17.0)	279 026 (17.2)	7674 (10.9)	3101 (19.4)		
2	385 172 (22.6)	365 889 (22.6)	15 362 (21.9)	3921 (24.6)		
≥3	1 032 788 (60.5)	976 608 (60.2)	47 251 (67.2)	8930 (56.0)		

Abbreviations: AHF, acute heart failure; HFPEF, heart failure with preserved ejection fraction; HFREF, heart failure with reduced ejection fraction; IMV, invasive mechanical ventilation; NIV, noninvasive ventilation.

Within the HFPEF group, patients treated with any respiratory support were younger, more likely to have chronic lung disease, chronic kidney disease, and to be obese. Within the HFREF group, those treated with invasive ventilation were younger. Those treated with NIV had higher prevalence of chronic lung disease and chronic kidney disease, diabetes, obesity, and smoking status. The NIV‐treated AHF patients were more likely to have chronic lung disease, obesity, and smoking status in both HFREF and HFPEF groups.

**Figure 2:**
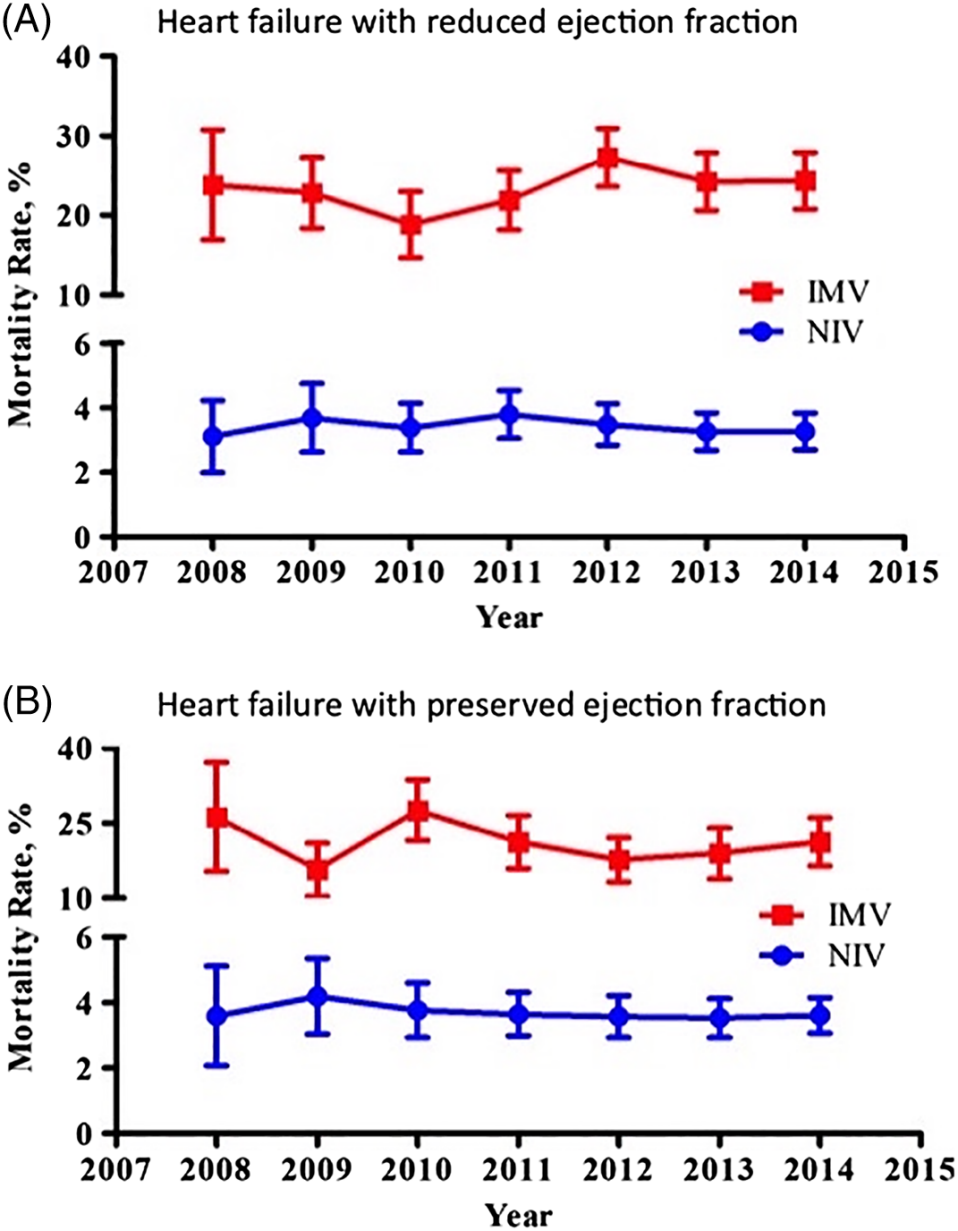
Age and sex adjusted mortality rates over time for heart failure with preserved ejection fraction (HFPEF) and heart failure with reduced ejection fraction (HFREF) ejection fraction patients treated with invasive and noninvasive ventilation. No statistically significant changes over time are observed (*P* values for trend: HFREF‐IMV *P* = 0.18, HFREF‐NIV *P* = 0.48, HFPEF‐IMV *P* = 0.18, HFPEF‐NIV *P* = 0.47)

Crude outcomes are shown in Table 2. Baseline rates of cardiogenic shock and in‐hospital arrest were lower in the HFPEF group compared to the HFREF group; however, requirement for respiratory support in both groups was associated with higher rates of cardiogenic shock and in‐hospital arrest. Both HFREF and HFPEF patients manifested high mortality if respiratory support was required. In‐hospital mortality for HFPEF patients treated with NIV was 4.3% and HFPEF treated with IMV 19.1%. In‐hospital mortality for HFREF patients treated with NIV was 4.7% and HFPEF treated with IMV 22.7%. Mortality has not changed over time for IMV or NIV treated patients in either group (Figure 2).

**Table 2:** Outcomes of AHF patients with preserved and reduced EF treated with respiratory support

(a) Select outcomes of patients with AHF due to HFPEF, NIS 2002‐2013						
					*P* value	
Characteristics	All (*n* = 1 331 236)	AHF without IMV or NIV (*n* = 1 244 078)	AHF with NIV (*n* = 79 571)	AHF with IMV (*n* = 7586)	NIV vs neither	IMV vs neither
Cardiogenic shock, No. (%)	3119 (0.2)	2228 (0.2)	369 (0.5)	521 (6.9)	<.001	<.001
In‐hospital arrest, No. (%)	1855 (0.1)	889 (0.07)	131 (0.2)	834 (11)	<.001	<.001
In‐hospital mortality, No. (%)	24 786 (1.9)	19 885 (1.6)	3455 (4.3)	1446 (19.1)	<.001	<.001
Hospital length of stay, mean (SD)	5.1 (5.0‐5.1)	5.0 (4.9‐5.0)	6.1 (6.0‐6.2)	10.0 (9.5‐10.6)	<.001	<.001

(b) Select outcomes of patients with AHF due to HFREF, NIS 2002‐2013						
					*P* value	
Characteristics	All (*n* = 1 707 762)	AHF without IMV or NIV (*n* = 1 621 523)	AHF with NIV (*n* = 70 287)	AHF with IMV (*n* = 15 952)	NIV vs neither	IMV vs neither
Cardiogenic shock, No. (%)	24 112 (1.4)	19 056 (1.2)	1483 (2.1)	3572 (22.4)	<.001	<.001
In‐hospital arrest, No. (%)	4674 (0.3)	2219 (0.1)	180 (0.3)	2275 (14)	<.001	<.001
In‐hospital mortality, No. (%)	35 070 (2.1)	28 123 (1.7)	3322 (4.7)	3625 (22.7)	<.001	<.001
Hospital length of stay, mean (SD)	5.1 (5.1‐5.2)	5.1 (5.0‐5.1)	5.7 (5.6‐5.8)	9.0 (8.7‐9.3)	<.001	<.001

Abbreviations: AHF, acute heart failure; HFPEF, heart failure with preserved ejection fraction; HFREF, heart failure with reduced ejection fraction; IMV, invasive mechanical ventilation; NIV, noninvasive ventilation.

### 3.3 Factors associated with respiratory support in HFPEF and HFREF

Factors associated with IMV and NIV use in HFPEF and HFREF are displayed in the supplemental table. For HFPEF patients in adjusted models, increasing age was associated with lower odds of IMV and NIV use, and comorbidities of chronic lung disease, diabetes, obesity, and chronic renal failure associated with higher odds of IMV and NIV use. Cardiogenic shock was associated with substantially higher odds of IMV use (OR 26.82, 95% CI 19.98‐36.01) and moderately increased odds of NIV use (OR 2.39 95% CI 1.85‐3.08). Among HFPEF patients, in‐hospital arrest was associated with very high odds of IMV (OR 139.99, 95% CI 108.9‐180.02) while in‐hospital arrest was not associated with NIV use.

For HFREF patients, increasing age was associated with lower odds of treatment with IMV but higher odds of treatment with NIV, female sex was associated with higher odds of support with both modalities. For HFREF patients, Hispanic and Asian‐Pacific Islander race were associated with higher odds of respiratory support as was chronic lung disease. Obesity was associated with higher odds of NIV but not IMV. Similar to HFPEF, cardiogenic shock was associated with substantially higher odds of IMV use and moderately increased odds of NIV use while in‐hospital arrest was associated with very high odds of IMV and not associated with NIV use.

Overall, chronic lung and kidney disease, diabetes, and obesity were associated with NIV use in both HFPEF and HFREF populations while cardiogenic shock and in hospital arrest as well as chronic lung disease were associated with IMV in both populations.

### 3.4 Mortality associated with respiratory support in HFPEF and HFREF

**Figure 3:**
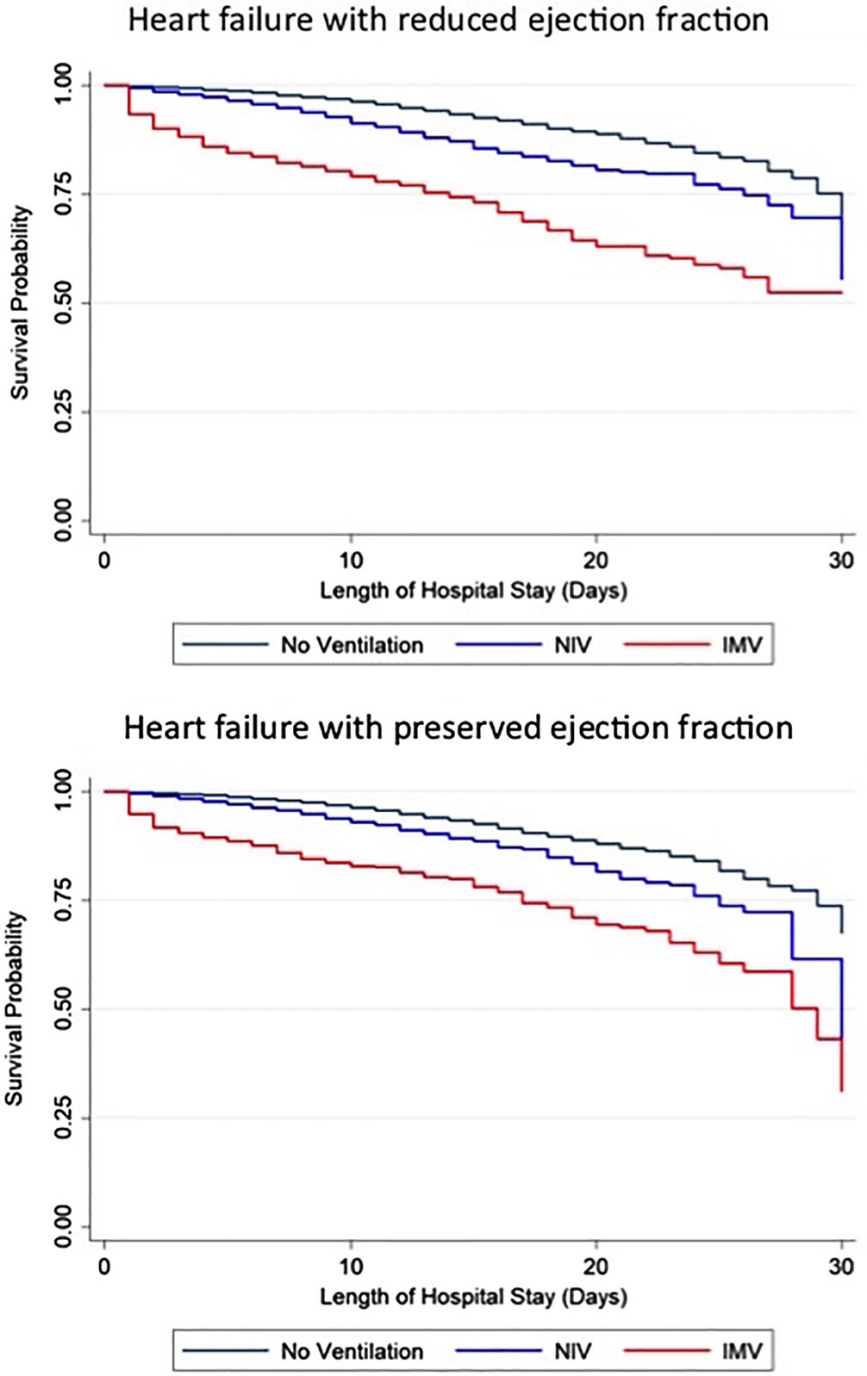
Kaplan‐Meier survival curves for acute heart failure patients with reduced and preserved ejection fraction treated with noninvasive ventilation, invasive ventilation, or neither modality

In HFPEF patients, NIV use was associated with 2.03‐fold increased risk for in hospital death compared to no respiratory support (HR 2.03, 95% CI 1.87‐2.21) and this increased risk persisted (HR 2.24 95% CI 2.05‐2.44) in a model adjusted for age, sex, race, comorbidity index, cardiogenic shock and in hospital arrest, chronic lung and kidney disease, diabetes and hospital size. In HFPEF patients, IMV use was associated with 4.06‐fold increased risk for in hospital death compared to no respiratory support (HR 4.06, 95% CI 3.53‐4.68) and this increased risk persisted (HR 2.85 95% CI 2.30‐3.53) in a model adjusted for age, sex, race, comorbidity index, cardiogenic shock and in hospital arrest, chronic lung and kidney disease, diabetes and hospital size. Kaplan‐Meier survival curves for NIV and IMV use compared to neither modality in both HFREF and HFPEF patients are shown in Figure 3.

This increased risk of in‐hospital mortality was similar among HFREF patients. In HFREF patients, NIV use was associated with 2.34‐fold increased risk for in hospital death compared to no respiratory support (HR 2.34, 95% CI 2.15‐2.55) and this increased risk persisted (HR 2.30 95% CI 2.11‐2.50) in adjusted models. IMV use was associated with 5.57‐fold increased risk for in hospital death compared to no respiratory support (HR 5.57, 95% CI 5.06‐6.13) and this increased risk persisted (HR 2.96 95% CI 2.57‐3.40) in adjusted models.

## 4 DISCUSSION

In our analysis of respiratory support in AHF stratified by HFPEF vs HFREF, we report several major findings. First, respiratory support with IMV and NIV is increasing in use in AHF hospitalizations with both HFPEF and HFREF, and HFPEF patients have higher rates of NIV use and lower rates of invasive ventilation use compared to HFREF. Patients with AHF who require support are at high risk for in‐hospital mortality irrespective of underlying EF and mortality is not declining over time. Finally, chronic lung disease and other patient comorbidities are associated with need for respiratory support in both populations as are cardiac comorbidities of cardiac arrest and cardiogenic shock. Our findings provide insights into important critical care complications in the HFPEF population which is important yet understudied to date.

### 4.1 Epidemiology of respiratory support in HFPEF vs HFREF

Our data support that respiratory support in AHF admissions is common irrespective of LVEF, and HFPEF patients are more likely to require NIV while HFREF patients slightly more likely to require IMV. Given the fact that over one million patients are hospitalized with heart failure each year nationally^26^ and that as many as half of these heart failure patients have preserved LVEF,^27^, ^28^, ^29^ our data confirm that many thousands of HFREF and HFPEF patients alike are exposed to positive pressure ventilation via NIV and IMV. Thus, both heart failure clinicians and acute care clinicians need to be aware of the rising use of respiratory support in their HFPEF and HFREF patients. Large registries of heart failure hospitalizations do not stratify respiratory support by LVEF,^30^, ^31^ therefore our results contribute epidemiologic data as to the scope of respiratory failure in HFPEF.

The greater requirement for NIV in HFPEF may reflect the burden of obesity and pulmonary vascular disease,^32^, ^33^ skeletal muscle dysfunction,^34^ and pulmonary diseases including COPD.^35^ For example, up to one‐third of HFPEF patients had COPD and half were obese in one study.^36^ Implications of this increasing burden of respiratory support in both HFPEF and HFREF include that (a) hospitals, heart failure programs, and clinicians providing care for these patients should maintain expertise in management of both forms of positive pressure ventilation, (b) thoughtful consideration of the appropriate ICU care and multidisciplinary care for patients with combined heart failure and respiratory failure will continue to be important,^37^, ^38^ possibly by involving a cardiac intensivist or critical care specialist, and 39 (c) the optimal means of respiratory support in cardiac patients is an unmet need in critical care research and our findings provide generalizable incidence rates for NIV and IMV use in both HFPEF and HFREF which will facilitate planning of intervention trials and sample size calculations.

### 4.2 Outcomes of respiratory support in HFPEF and HFREF

Requirement for NIV and IMV in AHF connotes poor prognosis, with high risk of in‐hospital death. While respiratory support per se does not cause adverse outcome, a conceptual model would be that heart failure patients who require respiratory support are at high risk for poor outcome due to the severity of underlying condition, due to comorbidities, and due to any risk of harm inherent in critical care support. Herein, we report that the adverse prognosis of heart failure complicated by respiratory failure is similar in both HFPEF and HFREF. While the overall death rate is slightly lower for HFPEF compared to HFREF,^27^, ^40^ our data suggest that all heart failure patients irrespective of LVEF are at high risk for in hospital mortality if the hospitalization includes respiratory support. Therefore, if AHF patients requires respiratory support, clinicians should not be reassured by a normal LVEF. It has been described that the profiles of congestion secondary to AHF are similar between HFPEF and HFREF patients^41^ supporting a common mechanism of respiratory failure leading to high patient risk. On the other hand, HFPEF patients have high rates of death due to noncardiovascular causes^5^ which supports a distinct but equivalently high‐risk mechanism of death. The NIS does not allow for ascertainment of cause‐specific mortality in our study. Although overall in‐hospital mortality in AHF hospitalization is declining,^42^ our results suggest that mortality has not declined among both HFPEF and HFREF patients who require respiratory support. Thus, these populations represent important groups to target future research, specialty expertise, and programmatic resources.

### 4.3 Factors associated with respiratory support in HFPEF and HFREF

Pulmonary disease was mildly associated with IMV use in both populations, however by far the strongest predictor of need for IMV was cardiac complications of cardiac arrest and cardiogenic shock, irrespective of LVEF. We also identify shared risk factors for NIV use across HFPEF and HFREF, including chronic lung and kidney disease, diabetes, and obesity. These shared risk factors for respiratory failure may point to a common mechanism of respiratory failure among AHF patients, irrespective of LVEF. Novel treatments to improve outcome in patients with HFPEF and respiratory failure are needed, and targeting comorbidities such as lung disease may be a promising treatment strategy.^43^, ^44^

### 4.4 Limitations

The main limitations of our study are related to the NIS as an administrative database. Thus, our study is unable to assess patient‐level factors such as specific LVEF or other echo parameters, labs assessed and drugs delivered during the hospital stay and disease‐specific cause of death. The NIS also does not provide an opportunity to evaluate postdischarge events or the specific mode of in‐hospital death. Classification of heart failure relies on accurate coding; however, the ICD‐9 diagnosis and procedure codes we use are consistent with prior literature and are well validated. Finally, as an observational risk factor analysis, our study can suggest associations but not demonstrate causality and as such, future studies in these patient populations are needed. Specifically, while our findings describe the association of respiratory support with outcome in HFPEF and HFREF, that is not to imply that patients who require respiratory support should not receive it. Rather, our findings should serve as an impetus to study the optimal mode of respiratory support and also provide general prognostic information for clinicians.

## 5 CONCLUSION

We demonstrate that the use of respiratory support with IMV and NIV is increasing in AHF hospitalizations with both HFPEF and HFREF. Requirement for respiratory support is associated with substantially higher risk for mortality irrespective of underlying HFPEF or HFREF, and mortality has not declined over time in either population. Studies assessing the mechanism of respiratory failure in HFPEF and HFREF and novel treatment strategies are needed to address these enlarging and high‐risk patient population.

### Conflict of interests

Dr Thomas S. Metkus reports personal fees from BestDoctors/TelaDoc Inc., personal fees from Oakstone/EBIX, personal fees from McGraw‐Hill publishing, outside the submitted work; Dr David A. Morrow reports grants and personal fees from Abbot Laboratories, grants from Amgen, grants and personal fees from AstraZeneca, grants from Daiichi Sankyo, grants from Eisai, grants and personal fees from GlaxoSmithKline, grants and personal fees from Merck, grants from Norvartis, grants and personal fees from Roche Diagnostics, grants from Medicines Company, grants from Quark Pharmaceuticals, personal fees from Verseon, personal fees from Peloton, personal fees from Bayer, personal fees from Aralez, personal fees from InCarda Therapeutics, outside the submitted work. Other authors have nothing to disclose.

## Supporting information


**Table S1** ICD‐9‐CM Codes used
**Table S2** (a) Association Between Select Factors and Type of Ventilation For patients with HFPEF, NIS 2002‐2013. (b) Association Between Select Factors and Type of Ventilation For patients with HFrEF, NIS 2002‐2013

## References

[1] Benjamin EJ , Virani SS , Callaway CW , et al. Heart disease and stroke Statistics‐2018 update: a report from the American Heart Association. Circulation. 2018;137(12):e67‐e492. 29386200 10.1161/CIR.0000000000000558

[2] Chioncel O , Mebazaa A , Harjola VP , et al. Clinical phenotypes and outcome of patients hospitalized for acute heart failure: the ESC heart failure long‐term registry. Eur J Heart Fail. 2017;19(10):1242‐1254. 28463462 10.1002/ejhf.890

[3] Dunlay SM , Roger VL , Redfield MM . Epidemiology of heart failure with preserved ejection fraction. Nat Rev Cardiol. 2017;14(10):591‐602. 28492288 10.1038/nrcardio.2017.65

[4] Sharma K , Kass DA . Heart failure with preserved ejection fraction: mechanisms, clinical features, and therapies. Circ Res. 2014;115(1):79‐96. 24951759 10.1161/CIRCRESAHA.115.302922 PMC4146618

[5] Vaduganathan M , Patel RB , Michel A , et al. Mode of death in heart failure with preserved ejection fraction. J Am Coll Cardiol. 2017;69(5):556‐569. 28153111 10.1016/j.jacc.2016.10.078

[6] Sharma A , Zhao X , Hammill BG , et al. Trends in noncardiovascular comorbidities among patients hospitalized for heart failure: insights from the get with the guidelines‐heart failure registry. Circ Heart Fail. 2018;11(6):e004646. 29793934 10.1161/CIRCHEARTFAILURE.117.004646

[7] Andrea R , Lopez‐Giraldo A , Falces C , et al. Pulmonary function predicts mortality and hospitalizations in outpatients with heart failure and preserved ejection fraction. Respir Med. 2018;134:124‐129. 29413499 10.1016/j.rmed.2017.12.004

[8] Farris SD , Moussavi‐Harami F , Stempien‐Otero A . Heart failure with preserved ejection fraction and skeletal muscle physiology. Heart Fail Rev. 2017;22(2):141‐148. 28255866 10.1007/s10741-017-9603-x PMC5487258

[9] Abdullah A , Eigbire G , Salama A , Wahab A , Nadkarni N , Alweis R . Relation of obstructive sleep apnea to risk of hospitalization in patients with heart failure and preserved ejection fraction from the National Inpatient Sample. Am J Cardiol. 2018;122(4):612‐615. 30205888 10.1016/j.amjcard.2018.04.052

[10] Eaton CB , Pettinger M , Rossouw J , et al. Risk factors for incident hospitalized heart failure with preserved versus reduced ejection fraction in a multiracial cohort of postmenopausal women. Circ Heart Fail. 2016;9(10):e002883. 27682440 10.1161/CIRCHEARTFAILURE.115.002883 PMC5111360

[11] Obokata M , Reddy YNV , Pislaru SV , Melenovsky V , Borlaug BA . Evidence supporting the existence of a distinct obese phenotype of heart failure with preserved ejection fraction. Circulation. 2017;136(1):6‐19. 28381470 10.1161/CIRCULATIONAHA.116.026807 PMC5501170

[12] Melenovsky V , Hwang SJ , Lin G , Redfield MM , Borlaug BA . Right heart dysfunction in heart failure with preserved ejection fraction. Eur Heart J. 2014;35(48):3452‐3462. 24875795 10.1093/eurheartj/ehu193 PMC4425842

[13] Parikh KS , Sharma K , Fiuzat M , et al. Heart failure with preserved ejection fraction expert panel report: current controversies and implications for clinical trials. JACC Heart Fail. 2018;6(8):619‐632. 30071950 10.1016/j.jchf.2018.06.008

[14] Gorter TM , van Veldhuisen DJ , Bauersachs J , et al. Right heart dysfunction and failure in heart failure with preserved ejection fraction: mechanisms and management. Position statement on behalf of the Heart Failure Association of the European Society of cardiology. Eur J Heart Fail. 2018;20(1):16‐37. 29044932 10.1002/ejhf.1029

[15] Metkus TS , Stephens RS , Schulman S , Hsu S , Morrow DA , Eid SM . Utilization and outcomes of early respiratory support in 6.5 million acute heart failure hospitalizations. Eur Heart J Qual Care Clin Outcomes. 2019;qcz030. [Epub ahead of print]. 10.1093/ehjqcco/qcz030 31225598

[16] Esteban A , Anzueto A , Frutos F , et al. Characteristics and outcomes in adult patients receiving mechanical ventilation: a 28‐day international study. JAMA. 2002;287(3):345‐355. 11790214 10.1001/jama.287.3.345

[17] Pinsky MR . Cardiovascular issues in respiratory care. Chest. 2005;128(5 suppl 2):592S‐597S. 10.1378/chest.128.5_suppl_2.592S 16306058

[18] Nava S , Hill N . Non‐invasive ventilation in acute respiratory failure. Lancet. 2009;374(9685):250‐259. 19616722 10.1016/S0140-6736(09)60496-7 PMC7138083

[19] Walkey AJ , Wiener RS . Use of noninvasive ventilation in patients with acute respiratory failure, 2000‐2009: a population‐based study. Ann Am Thorac Soc. 2013;10(1):10‐17. 23509327 10.1513/AnnalsATS.201206-034OC PMC3780971

[20] Vital FM , Ladeira MT , Atallah AN . Non‐invasive positive pressure ventilation (CPAP or bilevel NPPV) for cardiogenic pulmonary oedema. Cochrane Database Syst Rev. 2013;5:CD005351. 10.1002/14651858.CD005351.pub3 23728654

[21] Mehta AB , Syeda SN , Wiener RS , Walkey AJ . Epidemiological trends in invasive mechanical ventilation in the United States: a population‐based study. J Crit Care. 2015;30(6):1217‐1221. 26271686 10.1016/j.jcrc.2015.07.007 PMC4628853

[22] Saczynski JS , Andrade SE , Harrold LR , et al. A systematic review of validated methods for identifying heart failure using administrative data. Pharmacoepidemiol Drug Saf. 2012;21(suppl 1):129‐140. 22262599 10.1002/pds.2313 PMC3808171

[23] Khera R , Angraal S , Couch T , et al. Adherence to methodological standards in research using the National Inpatient Sample. JAMA. 2017;318(20):2011‐2018. 29183077 10.1001/jama.2017.17653 PMC5742631

[24] Kerlin MP , Weissman GE , Wonneberger KA , et al. Validation of administrative definitions of invasive mechanical ventilation across 30 intensive care units. Am J Respir Crit Care Med. 2016;194(12):1548‐1552. 27976941 10.1164/rccm.201605-0953LE PMC5215033

[25] Chandra D , Stamm JA , Taylor B , et al. Outcomes of noninvasive ventilation for acute exacerbations of chronic obstructive pulmonary disease in the United States, 1998‐2008. Am J Respir Crit Care Med. 2012;185(2):152‐159. 22016446 10.1164/rccm.201106-1094OC PMC3297087

[26] Ambrosy AP , Fonarow GC , Butler J , et al. The global health and economic burden of hospitalizations for heart failure: lessons learned from hospitalized heart failure registries. J Am Coll Cardiol. 2014;63(12):1123‐1133. 24491689 10.1016/j.jacc.2013.11.053

[27] Owan TE , Hodge DO , Herges RM , Jacobsen SJ , Roger VL , Redfield MM . Trends in prevalence and outcome of heart failure with preserved ejection fraction. N Engl J Med. 2006;355(3):251‐259. 16855265 10.1056/NEJMoa052256

[28] Goyal P , Almarzooq ZI , Horn EM , et al. Characteristics of hospitalizations for heart failure with preserved ejection fraction. Am J Med. 2016;129(6):635 e615‐635 e626. 10.1016/j.amjmed.2016.02.007 27215991

[29] Yancy CW , Lopatin M , Stevenson LW , de Marco T , Fonarow GC , ADHERE Scientific Advisory Committee and Investigators . Clinical presentation, management, and in‐hospital outcomes of patients admitted with acute decompensated heart failure with preserved systolic function: a report from the Acute Decompensated Heart Failure National Registry (ADHERE) database. J Am Coll Cardiol. 2006;47(1):76‐84. 16386668 10.1016/j.jacc.2005.09.022

[30] Adams KF Jr , Fonarow GC , Emerman CL , et al. Characteristics and outcomes of patients hospitalized for heart failure in the United States: rationale, design, and preliminary observations from the first 100,000 cases in the Acute Decompensated Heart Failure National Registry (ADHERE). Am Heart J. 2005;149(2):209‐216. 15846257 10.1016/j.ahj.2004.08.005

[31] Nieminen MS , Brutsaert D , Dickstein K , et al. EuroHeart Failure Survey II (EHFS II): a survey on hospitalized acute heart failure patients: description of population. Eur Heart J. 2006;27(22):2725‐2736. 17000631 10.1093/eurheartj/ehl193

[32] Dalos D , Mascherbauer J , Zotter‐Tufaro C , et al. Functional status, pulmonary artery pressure, and clinical outcomes in heart failure with preserved ejection fraction. J Am Coll Cardiol. 2016;68(2):189‐199. 27386773 10.1016/j.jacc.2016.04.052

[33] Kitzman DW , Shah SJ . The HFPEF obesity phenotype: the elephant in the room. J Am Coll Cardiol. 2016;68(2):200‐203. 27386774 10.1016/j.jacc.2016.05.019

[34] Lewis GA , Schelbert EB , Williams SG , et al. Biological phenotypes of heart failure with preserved ejection fraction. J Am Coll Cardiol. 2017;70(17):2186‐2200. 29050567 10.1016/j.jacc.2017.09.006

[35] Mentz RJ , Kelly JP , von Lueder TG , et al. Noncardiac comorbidities in heart failure with reduced versus preserved ejection fraction. J Am Coll Cardiol. 2014;64(21):2281‐2293. 25456761 10.1016/j.jacc.2014.08.036 PMC4254505

[36] Ather S , Chan W , Bozkurt B , et al. Impact of noncardiac comorbidities on morbidity and mortality in a predominantly male population with heart failure and preserved versus reduced ejection fraction. J Am Coll Cardiol. 2012;59(11):998‐1005. 22402071 10.1016/j.jacc.2011.11.040 PMC4687406

[37] Morrow DA , Fang JC , Fintel DJ , et al. Evolution of critical care cardiology: transformation of the cardiovascular intensive care unit and the emerging need for new medical staffing and training models: a scientific statement from the American Heart Association. Circulation. 2012;126(11):1408‐1428. 22893607 10.1161/CIR.0b013e31826890b0

[38] O'Malley RG , Olenchock B , Bohula‐May E , et al. Organization and staffing practices in US cardiac intensive care units: a survey on behalf of the American Heart Association writing group on the evolution of critical care cardiology. Eur Heart J Acute Cardiovasc Care. 2013;2(1):3‐8. 24062928 10.1177/2048872612472063 PMC3760580

[39] Katz JN , Minder M , Olenchock B , et al. The genesis, maturation, and future of critical care cardiology. J Am Coll Cardiol. 2016;68(1):67‐79. 27364053 10.1016/j.jacc.2016.04.036

[40] Lee DS , Gona P , Albano I , et al. A systematic assessment of causes of death after heart failure onset in the community: impact of age at death, time period, and left ventricular systolic dysfunction. Circ Heart Fail. 2011;4(1):36‐43. 21071547 10.1161/CIRCHEARTFAILURE.110.957480 PMC3243964

[41] Ambrosy AP , Bhatt AS , Gallup D , et al. Trajectory of congestion metrics by ejection fraction in patients with acute heart failure (from the heart failure network). Am J Cardiol. 2017;120(1):98‐105. 28479167 10.1016/j.amjcard.2017.03.249 PMC5471496

[42] Akintoye E , Briasoulis A , Egbe A , et al. National Trends in admission and in‐hospital mortality of patients with heart failure in the United States (2001–2014). J Am Heart Assoc. 2017;6(12):1‐14. 10.1161/JAHA.117.006955 PMC5779014 29187385

[43] Carter P , Lagan J , Fortune C , et al. Association of Cardiovascular Disease with Respiratory Disease. J Am Coll Cardiol. 2019;73(17):2166‐2177. 30846341 10.1016/j.jacc.2018.11.063

[44] Canepa M , Franssen FME , Olschewski H , et al. Diagnostic and therapeutic gaps in patients with heart failure and chronic obstructive pulmonary disease. JACC Heart Fail. 2019;7(10):823‐833. 31521680 10.1016/j.jchf.2019.05.009

