# Supplementary material for: Respiratory support in acute heart failure with preserved vs reduced ejection fraction
Source: Clin Cardiol. 2019 Dec 11;43(4):320–8. doi: 10.1002/clc.23317 (PMC7144479; doi:10.1002/clc.23317)
Supplement: Supplementary file 1 — Table S1 ICD‐9‐CM Codes used Table S2 (a) Association Between Select Factors and Type of Ventilation For patients with HFPEF, NIS 2002‐2013. (b) Association Between Select Factors and Type of Ventilation For patients with HFrEF, NIS 2002‐2013 [file CLC-43-320-s001.docx]

|  |  |
| --- | --- |
| **eTable 1: ICD-9-CM Codes used** |  |
| **Disease or procedure** | **ICD-9 diagnosis or procedure codes** |
| Acute heart failure with preserved ejection fraction | 428.31 or 428.33 |
| Acute heart failure with reduced ejection fraction | 428.21 or 428.23 |
| Non-invasive ventilation | 93.9 |
| Invasive mechanical ventilation | 96.7, 96.0 |
| Chronic pulmonary disease | CCS |
| Chronic kidney disease | CCS |
| Congestive heart failure | CCS |
| Diabetes, complicated | CCS |
| Diabetes, uncomplicated | CCS |
| Dyslipidemia | 272.x |
| Obesity | CCS |
| Peripheral vascular disease | CCS |
| Coronary artery disease | 414.0x |
| Smoking | 305.1x, V15.82 |
| Prior myocardial infarction | 412 |
| Prior percutaneous coronary intervention | V45.82 |
| Prior coronary artery bypass grafting | V45.81 |
| In-hospital cardiac arrest | 99.60, 99.63 |
| Cardiogenic shock | 785.51 |

eTable 2: Clinical factors associated with respiratory support in HFPEF and HFREF

| **Table 3a: Association Between Select Factors and Type of Ventilation For patients with HFPEF, NIS 2002-2013** | | | | | | | | |
| --- | --- | --- | --- | --- | --- | --- | --- | --- |
|  | | **Odds Ratio (95% CI)** | | | | | | |
|  | | **Invasive Mechanical Ventilation on Day 1** | | |  | **Non-Invasive Ventilation on Day 1** | | |
| **Factor** | | **Unadjusted** | **Adjusted** | ***P*Value** |  | **Unadjusted** | **Adjusted** | ***P*Value** |
| Age (every 10yrs >18) | | 0.76 (0.74-0.79) | 0.83 (0.79-0.86) | <0.001 |  | 0.88 (0.87-0.89) | 0.97 (0.96-0.99) | <0.001 |
| Gender | |  |  |  |  |  |  |  |
|  | Male | Reference | Reference |  |  | Reference | Reference |  |
|  | Female | 0.80 (0.72-0.89) | 0.87 (0.78-0.97) | 0.01 |  | 0.96 (0.93-1.0) | 0.98 (0.95-1.02) | 0.4 |
| Race | |  |  |  |  |  |  |  |
|  | White | Reference | Reference |  |  | Reference | Reference |  |
|  | Black | 2.07 (1.83-2.35) | 1.62 (1.41-1.87) | <0.001 |  | 1.24 (1.17-1.32) | 1.10 (1.04-1.17) | 0.001 |
|  | Hispanic | 2.00 (1.67-2.40) | 1.75 (1.41-1.87) | <0.001 |  | 1.28 (1.17-1.41) | 1.24 (1.13-1.37) | <0.001 |
|  | Asian/Pacific Islander | 2.48 (1.83-3.37) | 2.49 (1.79-3.48) | <0.001 |  | 1.45 (1.28-1.66) | 1.60 (1.41-1.83) | <0.001 |
|  | Native American | 0.75 (0.28-1.97) | 0.72 (0.27-1.87) | 0.5 |  | 1.05 (0.71-1.53) | 0.98 (0.66-1.44) | 0.9 |
| Charlson comorbidity index | |  |  |  |  |  |  |  |
|  | 1 | Reference | Reference |  |  | Reference | Reference |  |
|  | 2 | 0.96 (0.81-1.13) | 0.77 (0.63-0.93) | 0.007 |  | 1.64 (1.54-1.74) | 1.11 (1.04-1.18) | 0.003 |
|  | ≥3 | 0.93 (0.81-1.07) | 0.57 (0.45-0.72) | <0.001 |  | 1.76 (1.66-1.86) | 1.02 (0.95-1.10) | 0.6 |
| Chronic pulmonary disease | | 1.13 (1.02-1.25) | 1.32 (1.17-1.49) | <0.001 |  | 1.88 (1.82-1.95) | 1.79 (1.72-1.86) | <0.001 |
| Diabetes | | 1.25 (1.13-1.38) | 1.20 (1.05-1.37) | 0.007 |  | 1.35 (1.30-1.40) | 1.12 (1.08-1.17) | <0.001 |
| Obesity | | 1.57 (1.41-1.75) | 1.26 (1.12-1.43) | <0.001 |  | 2.04 (1.96-2.12) | 1.80 (1.72-1.87) | <0.001 |
| Chronic renal failure | | 1.12 (1.01-1.23) | 1.29 (1.11-1.50) | 0.001 |  | 1.13 (1.10-1.17) | 1.15 (1.10-1.20) | <0.001 |
| In-hospital arrest | | 160.11 (128.66-199.26) | 139.99 (108.86-180.02) | <0.001 |  | 1.20 (0.81-1.77) | 1.10 (0.73-1.66) | 0.6 |
| Cardiogenic Shock | | 37.52 (30.22-46.59) | 26.82 (19.98-36.01) | <0.001 |  | 2.12 (1.66-2.70) | 2.39 (1.85-3.08) | <0.001 |
|  |  |  |  |  |  |  |  |  |
| **Table 3b: Association Between Select Factors and Type of Ventilation For patients with HFrEF, NIS 2002-2013** | | | | | | | | |
|  | | **Odds Ratio (95% CI)** | | | | | | |
|  | | **Invasive Mechanical Ventilation on Day 1** | | |  | **Non-Invasive Ventilation on Day 1** | | |
| **Factor** | | **Unadjusted** | **Adjusted** | ***P*Value** |  | **Unadjusted** | **Adjusted** | ***P*Value** |
| Age (every 10yrs >18) | | 0.85 (0.83-0.86) | 0.88 (0.85-0.89) | <0.001 |  | 1.03 (1.02-1.04) | 1.09 (1.07-1.10) | <0.001 |
| Gender | |  |  |  |  |  |  |  |
|  | Male | Reference | Reference |  |  | Reference | Reference |  |
|  | Female | 1.10 (1.02-1.18) | 1.28 (1.19-1.38) | <0.001 |  | 1.14 (1.10-1.18) | 1.11 (1.07-1.15) | <0.001 |
| Race | |  |  |  |  |  |  |  |
|  | White | Reference | Reference |  |  | Reference | Reference |  |
|  | Black | 1.16 (1.06-1.26) | 0.98 (0.88-1.09) | 0.7 |  | 0.98 (0.92-1.04) | 1.04 (0.98-1.11) | 0.2 |
|  | Hispanic | 1.44 (1.26-1.65) | 1.31 (1.14-1.52) | <0.001 |  | 1.19 (1.08-1.30) | 1.27 (1.16-1.39) | <0.001 |
|  | Asian/Pacific Islander | 1.70 (1.32-2.19) | 1.50 (1.16-1.94) | 0.002 |  | 1.44 (1.26-1.64) | 1.63 (1.43-1.86) | <0.001 |
|  | Native American | 1.08 (0.65-1.80) | 0.89 (0.51-1.55) | 0.7 |  | 0.71 (0.50-1.0) | 0.74 (0.53-1.03) | 0.08 |
| Charlson comorbidity index | |  |  |  |  |  |  |  |
|  | 1 | Reference | Reference |  |  | Reference | Reference |  |
|  | 2 | 0.95 (0.85-1.06) | 1.01 (0.89-1.15) | 0.8 |  | 1.52 (1.43-1.62) | 1.12 (1.05-1.20) | 0.001 |
|  | ≥3 | 0.81 (0.73-0.89) | 0.94 (0.81-1.08) | 0.4 |  | 1.76 (1.66-1.87) | 1.16 (1.08-1.25) | <0.001 |
| Chronic pulmonary disease | | 1.04 (0.97-1.12) | 1.19 (1.09-1.29) | <0.001 |  | 1.91 (1.84-1.98) | 1.77 (1.75-1.89) | <0.001 |
| Diabetes | | 0.92 (0.85-0.99) | 1.01 (0.93-1.10) | 0.8 |  | 1.25 (1.21-1.30) | 1.11 (1.06-1.15) | <0.001 |
| Obesity | | 1.0 (0.90-1.10) | 0.87 (0.78-0.98) | 0.02 |  | 1.84 (1.76-1.92) | 1.88 (1.79-1.97) | <0.001 |
| Chronic renal failure | | 0.89 (0.82-0.95) | 0.94 (0.85-1.05) | 0.3 |  | 1.15 (1.11-1.19) | 1.07 (1.02-1.12) | 0.003 |
| In-hospital arrest | | 117.1 (101.8-134.8) | 93.7 (78.8-111.5) | <0.001 |  | 0.93 (0.67-1.29) | 0.86 (0.62-1.2) | 0.4 |
| Cardiogenic Shock | | 23.5 (21.1-26.08) | 18.8 (16.7-21.3) | <0.001 |  | 1.54 (1.36-1.73) | 1.77 (1.57-2.0) | <0.001 |
